# Cultural and religious structures influencing the use of maternal health services in Nigeria: a focused ethnographic research

**DOI:** 10.1186/s12978-024-01933-8

**Published:** 2024-12-18

**Authors:** Uchechi Clara Opara, Peace Njideka Iheanacho, Pammla Petrucka

**Affiliations:** 1 College of Nursing, University of Saskatchewan, Health Science Building - 1A10, 107 Wiggins Road, Box 6, Saskatoon, SK S7N 5E5 Canada; 2 Department of Nursing Sciences, University of Nigeria, Enugu Campus, Nsukka, Enugu State Nigeria

**Keywords:** Culture, Religion, Qualitative research, Maternal health, Maternal health services, PEN 3 cultural model

## Abstract

**Background:**

Cultural and religious structures encompass a set pattern of values, beliefs, systems and practices that define a community's behaviour and identity. These structures influence women's health-seeking behaviour and access to maternal health services, predisposing women to preventable maternal health complications. However, most maternal health policies have focused on biomedical strategies, with limited attention to women’s cultural challenges around childbirth. The overall aim of this paper is to provide a thick description and understanding of cultural and religious structures in Nigeria, their meaning and how they influence women’s use of maternal health services.

**Methods:**

Roper and Shapira’s (2000) focused ethnography comprising 189 h of observation of nine women from the third trimester to deliveries. Using purposive and snowballing techniques, 21 in-depth interviews and two focus group discussions comprising 13 women, were conducted in two Nigerian primary healthcare facilities in rural and urban area of Kogi State. Data was analyzed using the steps described by Roper and Shapira.

**Results:**

Using the PEN-3 cultural model, nine themes were generated. Positive factor, such as the language of communication, existential factor, such as religion, and negative factors, such as the use of prayer houses and lack of women’s autonomy, were either positive or negative enablers influencing women’s use of maternal health services. Additionally, women's perceptions, such as their dependency on God and reliance on cultural norms were significant factors that influence the use of maternal health services. We also found that the use of herbal medicine was a negative enabler of women’s access to facility care. At the same time, family support was also a positive and a negative nurturer that could influence how women use facility care. Finally, factors such as religion, Ibegwu, and male child syndrome were negative nurturers influencing women’s contraceptive use.

**Conclusion:**

Cultural and religious structures are significant factors that could promote or limit women’s use of maternal health services. Further studies are needed to understand culturally focused approaches to enhance women’s use of maternal health services in Nigeria.

**Supplementary Information:**

The online version contains supplementary material available at 10.1186/s12978-024-01933-8.

## Background

The use of maternal health services (MHS) such as antenatal, skilled attendance at birth, and post-natal care are critical steps towards reducing maternal mortality. Over the years, several strategies, such as the sustainable development goals (SDG #3:1), have been developed globally to reduce maternal mortality [1]. The SDG #3:1 aims to reduce maternal mortality to less than 70 per 100,000 live births and ensure a reduction of 140 per 100,000 live births in each country [2]. Despite the potential of this strategy to promote maternal health, women in many Sub-Saharan African (SSA) countries face challenges in accessing prenatal, birth, and postnatal care. Consequently, SSA countries contributed 70 percent of global maternal mortality in 2020 [3]. One factor that influences women's access to MHS is the cultural and religious structures within the context that shape decisions on facility care [4–6]. Given that most MHS strategies in SSA are biomedical-centric on promoting skilled birth attendance, limited attention has been paid to cultural and religious structures within communities that influence access to facility care.

Cultural and religious structures are collective norms, systems, practices, and beliefs that form a pattern of behaviour among a group of people [7, 8]. Such cultural and religious structures include language, arts, rituals, religion, customs, and other intergenerational cultural norms shared within a community [5, 8, 9]. In SSA, cultural and religious structures are further compounded due to cultural diversity comprising distinctive linguistic, religious, and cultural values and norms that play a crucial role in women's health-seeking behaviour around childbirth [10]. Thus, a critical exploration and understanding of these cultural and religious structures is essential in SSA given the cultural context and the emphasis on ‘ubuntu philosophy’ that emphasizes the significance of community and the interconnectedness of all individuals, promoting an environment of hospitality, justice, honesty and mutual support among individuals within a context [11, 12]. Consistent with the Ubuntu philosophy, pregnancy and childbirth in SSA heavily rely on cultural norms and day-to-day interaction among individuals within the community, which influence people's behaviour and shape decisions made around pregnancy and childbirth [13–17]. Consequently, it becomes challenging to meet the multifaceted maternal health issues in SSA with international and national maternal health policies that are mainly limited to scientific and technological strategies but lack relational principles.

Nigeria as an SSA country is also enmeshed in these cultural and religious structures, which also contributed significantly to the Nigerian high maternal mortality ratio of approximately 1047 per 100,000 live births, making Nigeria the third-highest contributor to maternal mortality globally in 2020 due to limited use of MHS [18]. Studies reveal that approximately 60 percent of women have access to antenatal care, while only 50 percent have access to facility birth [19, 20]. Low access to MHS also extends to the postnatal period, with only 35 percent access and with only four out of ten women having access to contraceptives in 2021 [21, 22]. Though several maternal health policies have been developed in Nigeria, such as the Integrated Maternal, Newborn and Child Health Strategies (IMNCH) and the Midwives Service Scheme (MSS) [23–25], both policies are biomedically focused on increasing skilled attendance. Medical and technological strategies are essential in maternal health, but their success remains hindered by the lack of cultural consideration development.

Studies reveal that cultural and religious structures influenced by existing cultural beliefs, practices, gendered roles, and social structures are significant issues that influence decisions around childbirth in Nigeria [26–29]. Additionally, religious norms and practices are critical issues linked to each other, which influence the uptake of MHS and maternal health outcomes [9, 30]. In Nigeria, the extent to which religion influences maternal health-seeking behaviours and decisions around childbirth differs among ethnicities and religious systems [31]. However, there is no known literature on religious norms and practices, their meaning and how they influence the use of MHS in Nigeria.

While there are several maternal health research in Nigeria, to our knowledge, there is no known research that focuses on understanding these cultural and religious structures and how they influence women’s use of MHS. Exploring these significant cultural and religious structures is critical to promoting understanding and provision of adequate holistic intervention tailored to the maternal health needs of communities. Therefore, this study aimed to understand these cultural and religious structures, what they mean to these women and how they influence the use of MHS using the PEN-3 cultural model as a framework for the findings. The PEN-3 cultural model is a mid-range theory used in several SSA countries and Nigeria to explore the influence of culture on health behaviour [32].

### The PEN-3 cultural model

The PEN-3 cultural model was developed to establish the role of culture in influencing health behaviours [33]. The model acts as an organizing framework that identifies an individual's or a community's cultural factors that impact a health intervention's planning. According to Iwelunmor et al. [34], the PEN-3 model comprises three cultural domains: cultural identity, relationships and expectations, and cultural empowerment [35]. Each domain is framed within three features; the cultural identity domain consists of a person, extended family, and the neighbourhood (PEN), whereas the relationships and expectations domain is made up of perceptions, enablers, and nurturers (PEN), and, finally, the cultural empowerment domain features positive, existential, and negative (PEN) factors [34]. Additionally, the PEN-3 cultural model consists of two phases, namely the intervention phase, which encapsulates the cultural identity domain where findings in research are implemented in the community [36]. The second phase (the assessment phase) focuses on cultural empowerment and the domain of relationships and expectations, where health issues are explored and assessed for possible future interventions [34]. This research will focus on the domain of cultural empowerment and the domain of relationships and expectations. The features of each domain will be further explained in the results section of the paper.

### Aim

To explore cultural and religious structures, their meanings and how they influence women’s use of MHS in Nigeria.

### Design

The study employed the focused ethnographic (FE) methodology described by Roper and Shapira [37], who emphasized the imperative to narrow research questions to study a distinct phenomenon within a population's subculture in a limited timeframe. FE in nursing research aims to study nursing as a cultural phenomenon and explore how diverse cultures incorporate health beliefs and practices within their context [37]. Research findings in FE seek to promote nursing practice and the integration of people's cultural norms in care to enhance health outcomes [37]. An FE is deemed appropriate as the methodology in this study as it enabled us to gain an in-depth understanding of the cultural and religious structures and norms among a selected ethnic group (Igalas) around childbirth that influence their use of MHS using our etic (participants) and emic (researchers) perspectives. As a critical part of FE, the authors also instituted passive and selective participant observation, which allowed exploring participant behaviour in the real world.

### Positionality

The first and second, authors acknowledge their positions as clinical nurse midwives and researchers among the Igalas in Kogi state, Nigeria. Both the first and second authors are Nigerians, have worked in numerous health facilities in Nigeria, and have lived among the Igalas for approximately 25 years. The first and second authors understand the Igala language and the pidgin English spoken by most of the population. The first author has practiced as a clinical nurse midwife in an obstetric department of a secondary health care facility among the Igalas in Kogi state for the past 25 years and has conducted several qualitative research among the Igalas. The second author is a university professor in Nigeria with extensive research and clinical experience in qualitative research. Finally, the third author is a Canadian with vast experience in maternal health research and research in Africa and other low-income contexts and is the overall supervisor of the study. The first author (who collected the data) was conscious of how she could influence the data collection and analysis process, given her exposure to the ethnic group. However, several approaches to reflexivity were employed to limit the researcher's influence.

### Reflexivity

The first author (who collected the data) employed the diffractive way of knowing, positioning her as an active participant in knowledge creation [38, 39], allowing for prolonged passive and selective observation of the contexts, participants, health workers, equipment, and practices. The researcher also ensured a deep immersion in the culture and continuously questioned her assumptions on issues taken for granted to promote a better understanding of the culture. Reflective memo-ing and continuous debriefing with members of the research team on a range of issues throughout the fieldwork and data analysis phase were significant in challenging personal interpretation and potential bias.

### Research setting

To promote a holistic understanding of cultural and religious structures among the Igalas, data was collected from two primary health facilities in the rural and urban areas of Olamaboro and Dekina local government of Kogi State, Nigeria. These primary health facilities were chosen as they offer comprehensive care ranging from antenatal care, care during birth, family planning, immunization, treatment of minor health issues, and provision of anti-retroviral medication. Community healthcare workers and other auxiliary workers manage both health facilities. The facility in the rural area is a 10-bed facility with a monthly average of eight women for antenatal care, 100 women for children’s immunization, 15 women for family planning, and two deliveries. The facility in the urban area is a 7-bed facility with a monthly average of antenatal care (23), immunization (39), family planning (7), deliveries (4), and provision of anti-retroviral medication (3).

### Sample/participants

The first author gained entrance to the primary health care facilities through the assistance of the gatekeepers in each facility. Most participants were selected using a purposive sampling technique, as the first author was able to speak to women on recruitment during antenatal and immunization health talks. Participation criteria for recruitment were displayed on the poster and flyers in each research setting and by the gatekeepers, who were well-informed about the study. Following the presentation, women interested in the study contacted the first author privately and were recruited after gaining oral permission from their husbands. The researcher privately met with these women to finalize the recruitment process and ensure women sign the informed consent form for selective observation, in-depth interviews and focus group discussions. The snowballing technique was also employed when recruited participants invited other women who met the inclusion criteria. The participants included in the study were Igala women aged 18–43 years who were either pregnant or had given birth at home or in a primary health care facility in Kogi state within the last 12 months. See Table 1 for the demographic characteristics of women included in the study.

**Table 1:** Participants demographic characteristics

Location	Number	Age	Marital status	Parity	Educational qualifications	Religion	Occupation
Rural Area Participant Observation (RA/PO),	n = 5	20–33	Married	1–4	High school diploma (5)	Christians (2) Muslims (3)	Housewives (5)
Urban Area Participant Observation (UA/PO),	n = 4	24–27	Married	1–4	Nil education (2) High school diploma (2)	Muslim (3) Christians (1)	Housewives (4)
Rural Area In-depth Interviews (RA/IDI),	n = 11	20–43	Married	2–6	Nil education (1) High school diploma (5) Associate degree (4) Bachelor’s degree (1)	Muslim (6) Christians (5)	Housewives (8) Business (1) Teaching (1) Nursing (1)
Urban Area In-depth Interviews (UA/IDI),	n = 10	19–40	Married	1–5	High school diploma (5) Associate degree (4) Bachelor’s degree (1)	Muslim (6) Christians (4)	Housewives (8) Public servant (1) Teaching (1)
Rural Area/Focus Group Discussions (RA/FGD),	n = 6	23–42	Married	1–4	High school diploma (2) Associate degree (3) Bachelor’s degree (1)	Muslim (3) Christians (3)	Business (3) Teaching (3)
Urban Area/Focus Group Discussions (UA/FGD)	n = 7	18–43	Married	1–5	High school diploma (4) Associate degree (2) Bachelor’s degree (1)	Muslim (6) Christians (1)	Business (3) Teaching (1) Public servant (2) Farmer (1)

Rural Area Participant Observation -(RA/PO), Urban Area Participant Observation- (UA/PO), Rural Area In-depth Interviews –(RA/IDI), Urban Area In-depth Interviews -(UA/IDI), Rural Area Focus Group Discussions -(RA/FGD), Urban Area Focus Group Discussions –(UA/FGD)

### Data collection

#### Observations

Data was collected for four months, from August 2023 to November 2023, with over 189 h of participant observation. At the beginning of the fieldwork, the first author began with passive observation in both facilities to promote an understanding of the research setting and the flow of patients without interaction with individuals. Observation flyers were also posted at strategic points in each health facility to create awareness of participant observations. Next, selective participant observation was instituted, which allowed the researcher to interact with the participants and the health workers in each facility. The selective participant observation also allowed the researcher to follow through with five women in the last trimester of pregnancy and four women from the last trimester of pregnancy to birth. During the observation, the researcher noted the language and approach to communication, men’s engagement in maternal health, health teachings, health services provided during births, approach to decision-making, attitude to family planning, cultural issues related to pregnancy, labour and birth, non-verbal communication, and health workers attitude to women’s cultural preferences. Participant observation lasted for approximately 4–5 h every day but was longer on days the researcher was observing pregnant women, labour, and birth cases. Observations were conducted mainly in the antenatal outpatient setting, during patient consultation with health workers in consulting rooms, and in the delivery room during labour and births. Both approaches to observation were guided by an observation guide that provided prompts on observed events, location, dates, and time, which were recorded in the field note. Data from field notes were converted into Word document^™^ within 12 h after the observation and were assigned numbers, as reflected in this paper. Data analysis in this paper commenced during the fieldwork so that initial findings are integrated into observation and interviews. During fieldwork, the first author also gathered information from the flyers, posters, and documents publicly available in both facilities.

#### Interviews and focus group discussions

The recruitment for in-depth interviews and focus group discussions ran concurrently with selective participant observations. The first author moderated all interviews and facilitated all focus group discussions using semi-structured interviews conducted between August 2023 and November 2023, primarily in health facilities and safe places for the researcher and participants. A total of 21 in-depth interviews were conducted with eleven women in the rural area and 10 women in the urban area who recently gave birth in primary health facilities, which lasted between 30 and 50 min. Participants were encouraged to provide an account of their experience and the meanings of cultural and religious structures that influence their use of MHS. An interview guide was developed for this study and validated by two authors to enhance the clarity of each question and its relevance to the research purpose. The interview guide's content and structure were informed by a literature review on cultural issues around childbirth in SSA countries, generated themes in interviews and factors observed from the study related to the research question. The interview guide was pretested on three women of the same characteristics as participants to enhance the clarity of the questions. However, the generated data was not included in the study. The main questions asked include: Can you tell me about health services provided for you around childbirth? What language do health workers use to communicate with you during pregnancy and childbirth? What role does your religion play that influences your use of facility care? Can you tell me about prayer houses and how women use them around childbirth? How do you make decisions related to pregnancy and childbirth in your home? What is your opinion about cultural beliefs and practices around pregnancy and childbirth in this community? How do you see God in your situation? Can you tell me about herbal concoctions and medicine used by you or women in this community? What role do your husband and family play around pregnancy and childbirth? What cultural factors in your community influence your use of contraceptives?

Two focus group discussions were conducted with six women in the rural area and seven women in the urban area who were pregnant or had recently given birth, using a guide that built-in generated themes from observation and interviews. Focus group discussions enhanced the triangulation of findings in both the interviews and observations, which lasted an average of one hour and fifteen minutes. Oral consent was obtained from participants to audio record the interviews and focus group discussions. Interviews and focus group discussions were conducted mainly in English and a few in pidgin English (an Indigenous language of communication derived from the English language), which is spoken generally by the population and by the first author. A back translation of all interview guides, consent and recruitment documents was conducted [40] by the first author, who understands both English and pidgin English and was checked and validated by the second author. All interviews and focus group discussions were conducted privately with only the participants and the first author.

In this study, reaching saturation was not spontaneous. The first author gained insight from preliminary observations to expand on new observations. In addition, insights and knowledge drawn from previous observations and interviews were used to expand on future interviews and focus group discussions. Data collection was refined to accommodate emerging themes until saturation was achieved. Thus, data saturation was reached when emerging themes were supported with substantive data with no emerging new or contradictory information.

### Data analysis

Data analysis was iterative, going back and forth until the researchers gained a thick description of the phenomenon under review. All data from interviews, focus group discussions, policy documents, posters, reflective memo and field notes were transcribed verbatim. However, a back translation was done for a few interviews and focus group discussions conducted in pidgin English, which was also included in the analysis. The five steps of data analysis described by Roper and Shapira [37]: (a) coding for descriptive labels, (b) sorting for patterns, (c) identification of outliers or negative cases, (d) generalizing with constructs and theories, and (e) memoing and reflective remarks were employed in this study. However, data analysis was not chronological as the researchers kept moving back and forth until a thick description and interpretation were achieved [37, 41].

In the first step of coding for descriptive labels, the transcripts were read line by line repeatedly by the authors, who met regularly to understand the broad themes and patterns. The authors also deliberated regularly on the data coding to understand the cultural and religious structures, their meanings and how they influence women’s use of MHS from each participant transcript. Coding was done manually, which allowed immersion in the data and generated themes and subthemes. In the second step of sorting for patterns, the authors repeatedly read the transcripts to understand subthemes or categories of patterns occurring in each theme [37]. The identified themes were then compared with the interviews and observations and discussed among the authors as patterns continued to emerge, requiring more exploration of emerging themes. In the third step of identification of an outlier, the researchers sought to understand themes not connected to the study but are vital factors influencing cultural factors. In the fourth step of generalizing of constructs and theories [37], the researchers sought to understand how the findings relate to existing literature. The last step of memoing and remarks [37] allowed for continued reflection and transparency, enhanced by the reflective notes documented throughout the research process. See Table 2 for themes, codes, and excerpts of the study findings.

**Table 2:** Themes, codes and excerpts

Themes	Code	Excerpts
Language of communication	Health information in Igala language Interpreter of health information	*The things that influence me, that make me understand is that, they didn’t use any language apart from that Igala. It makes me understand what they said* *so if you understand English, they will speak English to you; if you don’t understand, they will speak Igala.. someone will be there to interpret it for you)*
Influence of religion	Prayer and financial support Prayer and Robutu	*my pastor prayed for me and other women that gave birth. and also gave us money* *When I am pregnant, we usually go to Imam to pray for us, he will write that thing* (robutu)* on a wood, then we will wash it use water to wash it down*
Influence of prayer houses	Prayers and protection Facility care and break in protection Selling of prayer artifacts and demand for money	*I was in the prayer house that she said I have to spent eeh 14 days there, so that the pregnancy will be safe* *They prayed for me and anointed me with oil and told me not to go to the maternity because my mother-in-law was after my life* *you will be bringing the money for them (prayer houses), and they will be using it to take care of their own family*
Women’s lack of autonomy	Husband makes decisions Other family member make decisions The stigma attached to women making decisions	*Because he is the head of the house, if you are going somewhere and he says you are not going there, then you will sit down* *You know, we believe here that every day you are married to your husband, he is not the one to take the whole decision* *They will say the woman is a strong woman, they will say the woman married the man, not the man marrying the woman;*
Seeking belonginess	It is our tradition	*it means a lot, … because that is our belief, that is tradition, and it has to be respected*
Relying on the supremacy of god	God can do all things	*We see God through our prayer.. because some of us see God as our helper…. the baby in our womb, we don’t know how the baby is. It is only God that know*
Herbal medicine	Types of herbal medicine and use Combining herbal and Western medicine Local medicine has no dosage	*they call one ‘ogwu ija’ ….so that the baby will not be big in your tummy and comes down with…..different … sickness* *This herbal medicine is very important because they will ask you to take these herbal medicines and not to concentrate on hospital drugs alone* *Local medicine doesn’t have dose and if you take overdose, it will affect you. Some it will make you to be vomiting or having hot temperature or some other things*
Family support	My husband provides the money I don’t want my husband at antenatal No extended family	*My husband provides all the money that I spend here during my antenatal and delivery* *So, he will not give you the amount that you need again because he already knows what they are doing in that place* *I don’t like it. Like my own, they came, but I was not okay with it*
Factors influencing contraceptive use	Ibegwu *Male Child Syndrome* Our religion	*they said that a married woman, is not good to go and do family planning, because of those Ibegwu (ancestors)…because the thing (ancestors) leads to the man’s death* *When you don’t have a male child, you can’t go on family planning* *Islamically, they will be preaching against it (modern contraceptives),.. because they believe doing that (using modern contraceptives) is as if you are also committing an abortion indirectly*

### Trustworthiness

Trustworthiness follows Lincoln and Guba's criteria of credibility, transferability, dependability, and confirmability [42]. To meet the credibility criterion, the research instruments were piloted with three women of similar characteristics to the participants before data collection to enhance the validity and appropriateness of the instruments to the research question. These results were not included in the final document review. Additionally, there was consistent debriefing of the findings with other co-authors to enhance trustworthiness. The first author also member-checked transcripts with participants to reduce error and enhance transparency. To enhance the transferability criterion, we employed the purposive and snowballing technique, which ensured that participants were selected based on their experience with the phenomena of interest, which enriched the data. To enhance the criterion of dependability, we provided a detailed description of the data collection approach and ensured that members of the research group carried out coding to reduce bias. To ensure the criterion of confirmability, a detailed audit trail, reflective note, and several data triangulation approaches, such as field notes, observations and focus group discussions, were employed to enhance the confirmability of research findings and a rich study.

## Results

**Fig. 1:**
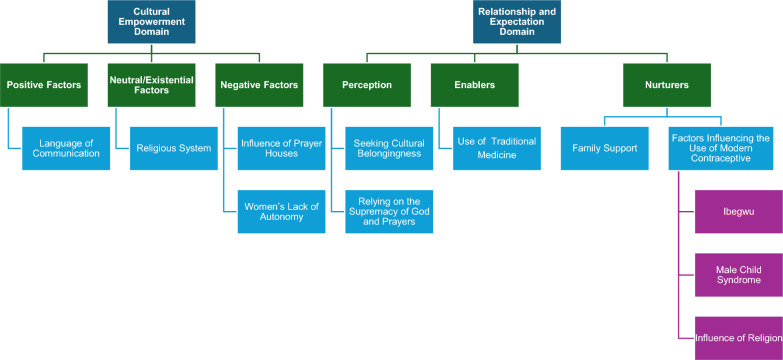
Summary of the themes and subthemes using the PEN-3 cultural model

A total of 43 pregnant and nursing women, aged between 18 and 43 years, with parity ranging from one to six of diverse demographic characteristics, participated in the study. Using the PEN-3 framework, themes from the findings in the cultural empowerment domain include positive factors such as the system of communication. A positive factor, language of communication, was identified in this domain as a positive factor that influenced women’s use of MHS. An existential factor under this domain identified was religion. Additionally, negative factors that influence women’s use of MHS identified in this domain are prayer houses and lack of women’s autonomy. In the relationship and expectation domain, seeking belongingness and the belief in the supremacy of God were the perceptions identified in the study. A negative enabler identified is the use of traditional medicine. At the same time, nurturers in the findings include family support and factors influencing the use of contraceptives. See Fig. 1 for a Summary of the Themes and Subthemes Using the PEN-3 Cultural Model.

### Cultural empowerment domain

In this domain, the authors first explored cultural and religious structures within the community that positively influence the use of MHS, and then those that are existential (harmless) are acknowledged. Finally, cultural and religious structures that are harmful or act as barriers to women’s use of MHS are explored.

#### Positive factor

Positive factors are those cultural systems or structures that promote women’s use of MHS.

#### Language of communication

Most women narrated that they grow, learn, and interact through the Indigenous Igala language, which allows them to communicate effectively within their context. In this study, women identified the use of Indigenous oral communication using the Igala language as a positive factor that influenced their use of MHS. Women recalled that the use of the Igala language contributed immensely to their oral expression, engagement in health education, understanding of health information and a reason for their continued use of the primary health facility of their choice.

*They teach us in Igala, it makes us understand what they are teaching us very well… it is interesting…the Igala that they are teaching us will make us come back another day again (RA/IDI/07).*

*Because I am Igala by tribe and I was born an Igala, my mum and dad are Igala by tribe, so its sweet in my heart and it makes me very happy. The things that influence me, that make me understand is that, they didn’t use any language apart from that Igala. It makes me understand what they said (RA/IDI/08).*

*Yes, the dialect (Igala language) is beneficial; for instance, now, if they are not feeling fine, either the head or the stomach, they will explain to them, but if they tell them to speak English, none of them would be able to speak English. If they speak their dialect, they will understand it better than English….since they understand what they taught them through that dialect, the thing will motivate them; next time, they will rush to that hospital, to that health centre (UA/IDI/09).*

*Normally, you know some people do not understand English, so if you understand English, they will speak English to you; if you don’t understand, they will speak Igala.. someone will be there to interpret it for you (UA/IDI/07).*

Participants also recounted that in most health facilities where health information is provided in English, health workers also take time to interpret the information in Igala and other languages for women who do not understand English to promote an in-depth understanding and engagement in maternal health.

#### Neutral/existential factor

Neutral behaviours are those cultural and religious structures in the community that do not significantly contribute to women’s use of MHS.

#### Religious system

Findings from the study showed that the most dominant religious systems in the communities are Christianity and Islamic religion. Results from the study show that both religious systems play diverse roles around childbirth. Some of the roles played by these religious systems could be significant support during pregnancy and birth. However, such support may not necessarily enhance the use of MHS. For Christians, such support ranges from prayers to monetary support at birth, as these women recount.

*When I gave birth to my first issue, my pastor came down to the hospital to come and see me and prayed for me and other women that gave birth. and also gave us money and, helped me, transported me from the hospital back to my house (UA/FGD/01).*

*when you are pregnant it is not a spiritual matter. You only go to your pastor when you need prayers. It is just that from time to time, maybe in the church, the pastor will pray for all pregnant women, and then when you are in labour, they will even come physically with their wife to pray with you (RA/IDI/01).*

The Islamic participants in the study also noted support from their religious leaders or Imams, ranging from prayers to the provision of Rubutu. Rubutu is the verses of the holy Quaran or spiritual prayer book, written on a board, which are then washed off and given to women to drink and rub on their private parts, believed to prevent pregnancy complications and evil attacks and enhance speedy birth. Rubutu is taken regularly to achieve an uneventful birth.

*When I am pregnant, we usually go to Imam to pray for us, he will write that thing (robutu) on a wood, then we will wash it use water to wash it down. He will wash it down and give us to drink, you will use it to wash your belly down to your front…whenever you use it to wash the private part it will make it to open so that you can deliver freely (RA/IDI/07).*

Women narrate that the support provided for women by these religious leaders is crucial and supports the cultural and spiritual expectations of women in communities. However, these supports do not influence or enhance women’s use of facility care.

#### Negative factors

Negative factors identified in the study are significant cultural and religious structures that influence the non-use of facility care or MHS. These negative factors are embedded in communities and are mostly viewed as protective factors that could enhance maternal health outcomes. Moreover, most women tenaciously hold on to these factors and are unaware of their negative consequences. The influence of prayer houses and lack of autonomy were the negative cultural and religious structures found in this study.

#### Influence of prayer houses

Prayer houses, also synonymous with healing homes and prayer ministries, are non-denominational, privately owned prayer homes which have proliferated not just in Igala land but throughout Nigeria. Women narrated that most prayer house owners claim they have the power to hear from God and to reverse health issues, including maternal health challenges. Given the cultural context, most people believe that pregnancy is a time when women are vulnerable to attacks from evil forces, which intensify the need to seek protection from the prayer houses. In most instances, women are made to undergo rituals and hibernation in prayer houses for weeks or months, even in the face of maternal health complications.

*I was in the prayer house that she said I have to spent 14 days there, so that the pregnancy will be safe so that she will be helping me to pray, I was there when I had the last miscarriage (RA/IDI/04).*

Some women are even told that stepping out of the prayer house to seek facility care would terminate the protection they have and predispose them to attacks from their enemies. The fear of losing their lives and their babies keeps them bound in such prayer houses, limiting their access to facility care, which could predispose them to maternal health complications.

*They prayed for me and, anointed me with oil and told me not to go to the maternity because my mother-in-law was after my life. They said the moment I go to the maternity, the protection will be broken, and I will either die or my baby will die. I was afraid (Observation 1).*

*You go to the prayer house a times they will tell you that you should not go to the hospital, that they will give you anointing, that they will give you water, that you should take it that it will help you (RA/IDI/07).*

Most women complain that they have become banks of many prayer houses, as they demand money ceaselessly from them. Additionally, women are presented with prayers and made to go through several rituals to ensure that their prayers are answered. These rituals mostly involve use of prayer artifacts such as candles, anointing oil, handkerchiefs, sand, salt and holy water, which are sold to these women in most prayer houses. Consequently, the money that could have been used for facility care is spent in these prayer houses, limiting women's access to facility care, given that facility care in most Nigerian communities is paid out of pocket.

*They give us olive oil and, in some places, they pack sand for us, that sand we put it in water, some are salt and some are different medicine… and you will be bringing the money for them (prayer houses), and they will be using it to take care of their own family; you will be suffering and be looking helter-skelter for money (RA/IDI/03).*

Even with all the promises from prayer houses, women complain that they do not see any change in their circumstances due to the high number of maternal health complications that are seen in most prayer houses. However, many women still go from one prayer house to another, looking for miracles, mainly when pregnancies are associated with complications. For some women, going to prayer houses is a multiplication of issues as they believe that many prayer house owners are possessed by evil spirits, which is then transferred to people who attend such places, which is the reason many say they do not access prayer houses for spiritual help around childbirth.

#### Women’s lack of autonomy

Lack of autonomy was one of the themes that evolved in the study, reflecting that patriarchy is a dominant system in Nigeria, where men control affairs in the home, including issues related to MHS use. Issues of patriarchy are openly related to couples during marriage where they are told that, henceforth, the man takes charge of decisions in the home. Consequently, men, in some cases, dictate if, when, how, and where women should seek facility care around childbirth, though most of the men do not engage in maternal health and do not have much knowledge or information about maternal health.

*You can’t make decisions by yourself except your husband. Because he is the head of the house, if you are going somewhere and he says you are not going there, then you will sit down, you will not go to that place (UA/IDI/04).*

In these contexts, apart from husbands, several other people, such as family members, are also involved in decision-making related to women’s use of MHS. Such an approach to decision-making is believed to be crucial in Igala land because if a complication or death occurs without prior notice to the woman’s family of an existing emergency, the husband would be required to provide explanations to the wife’s family. Consequently, decisions need to be obtained from the family of the woman in emergencies before such a woman could be transferred to a health facility for emergency care, delaying access to emergency health services that predispose women to maternal and fetal health complications.

*This is my first time coming to this hospital, there is where I use to go, my husband said I should stop going there, I should come here even my mother supported here, my dad also supported here that I should come here that they normally take care of pregnant women here. That is why I’m here (UA/IDI/ 07).*

*You know, we believe here that every day you are married to your husband, he is not the one to take the whole decision. Like if a woman is to be operated on, some husbands, no matter how emergent the situation is, they will ask the woman to wait. Let’s inform your parents and see what they will say. And some parents will say bring back the woman to us. It happened to one of my friends. The husband was willing to sign but the father said bring her home (RA/IDI/01).*

Many women interviewed stated that the cultural stigma attached to women making decisions limits them from taking such challenging steps. For example, women who make decisions in their homes are seen as stubborn and taking the place of their husbands. Such women are conceived as having charmed their husbands to occupy such a position of authority. The husband is also seen as a worthless man who is ruled and controlled by a woman.

*They will say the woman is a strong woman, they will say the woman married the man, not the man marrying the woman; they will say it is the woman that is the head of the house. They will be scared of that woman; they will say you should avoid the woman that the woman is the husband, and the husband is the wife (RA/IDI/09).*

Though a few women still maintained that decisions on the use of MHS are taken jointly by the couple, women narrated that, in some cases, decisions are delayed, especially during emergencies, which could delay maternal health access and result in preventable maternal and fetal health complications.

### The relationship and expectation domain

In this domain, we explored women’s perceptions and views of some significant cultural issues that influence their use of MHS. Additionally, some cultural and religious structures within the communities that influence women’s use of MHS were explored. We also explored some intergenerational traditions as well as the family, kin and friends within these communities that continuously act as a force to enable, nurture or hinder women’s use of MHS.

#### Perception

Women’s perceptions that were found in this study were the non-observable beliefs and values that influence their use of MHS such as seeking cultural belongingness and relying on the supremacy of God.

#### Seeking cultural belonginess

Many women narrated that they ascribe tenaciously to intergenerational practices to have a deep connection and link with their environment, social groups, and individuals with whom they share a close relationship. Such a connection is a fundamental human need as they believe that disconnection from the culture would mean that they were not rightfully married into the community or that they are strangers or outsiders. Women were also afraid that disconnection from traditional norms in the community could result in death and/or calamity in the family.

*It means a lot to me because…we take an oath that we will remain for our husband alone till death do us part. So that is an oath, so we respect the rules and regulations guiding our culture (RA/IDI/02).*

*…it means a lot, … because that is our belief, that is tradition, and it has to be respected. Because once you don’t believe in it you might end up dying young (RA/IDI/07).*

Some of the women interviewed had contrary views stating that such connections to traditional norms and practices were insignificant as they were brought up differently. Additionally, some participants expressed that such an era of holding on to cultural values and norms to prove your rightful belongingness to the community has passed and women need to embrace the new way of life by seeking assistance from health facilities to limit maternal mortality.

*To me, I didn’t believe in culture, I believe in almighty God. I will not be scared of any culture, I don’t believe in culture and culture didn’t see me and I didn’t see culture. I believe in God (RA/IDI/10).*

*Hm, actually some of my friends like me, I don’t observe them because I believe that era is passed – we are now in modern era and we should access modern facilities (RA/IDI/01).*

Many women stated that the need to maintain peace and acceptance in the family and community keeps them from openly expressing their feelings and walking in disobedience to cultural expectations and demands. Most pains are borne in their hearts and find expression as they carry out their daily activities in the home.

#### Relying on the supremacy of god and prayers

“What God cannot do does not exist” was a phrase that was frequently stated by participants in this study. Several of the women believed in the supremacy of God, implying that God has the sole authority and power over their lives and that of the baby. Many women in the study believe that God put the baby in the womb and so is able to protect and keep the baby until it pleases God to bring the baby out. Consequently, many women mentioned that they depend on prayers and God to see them through pregnancy and birth. They further narrate that, while health facilities exist, God alone has the final decision of maternal health outcome.

*We see God through our prayer.. because some of us see God as our helper…. the baby in our womb, we don’t know how the baby is. It is only God that know how the baby is in our womb, and again, that’s why I also call him, so God is our helper, He is the one that helped this pregnancy…. Prayer is the number one thing. I don’t joke with prayer. So, my advice to other women is that they should hold on to God when they are pregnant because that is when they need God the most (UA/IDI/01).*

Women, irrespective of their religion, also narrated that their belief and reliance on God gives them peace of mind and strength throughout the journey of pregnancy and childbirth. Women narrate that their faith in God is activated through worship and prayers to God, who alone has the final say in their condition.

#### Enablers

We found that the alternative health system was a critical healthcare system that was used by many women in the communities where the data was collected. Many women use traditional birth attendants and herbalists who provide them with traditional medicines during pregnancy and birth.

#### Use of traditional medicine

Women narrate that the use of alternative medicine in Igala land is an intergenerational practice that has been proven to be effective in relieving and treating minor and major maternal health issues. Many women assert that traditional medicine can easily be obtained from herbalists and traditional birth attendants who have an in-depth knowledge of herbal medicine. Women also claim that some traditional medicines are used commonly as spices and vegetables in the community. Women narrated the different types of herbal medicine they use and what each one does for them. Most of the traditional medicine are cooked or pounded to extract the liquid before they can be used by women.

*one they call ‘ogwu amaro’ if they give you that one it will protect the baby in your womb then if the time when you are in labour you …will be strong and you will deliver…. It will protect the mother too, if you to want to pee you will pee freely… you will not feel pain (UA/IDI/04)*

*If you are pregnant you will take native medicine, some of such native medicine or herbs are used to treat conditions such as ‘ofu’ (hemorrhoid), ‘ija’ (fibroid), and ‘utebie’ (prevent the blood coming out from your babies cord from turning black), they will cut these native medicines from the bush and we will cook and drink it so that it will help us during pregnancy (RA/IDI/06).*

*for the Igala people they call one ‘ogwu ija’ ….so that the baby will not be big in your tummy and comes down with…..different … sickness (UA/IDI/ 08).*

*whenever you go to the hospital at times they will tell you you don’t have blood. We have herb medicine, if you see it, it is like blood, They call it ‘ogwu ebie’. It is like blood, if you cook it, if any body tell you this is not blood, you will not believe it. We take that whenever the nurse say that we don’t have blood…it works (RA/IDI/ 07).*

*The medicines is ‘Ukpokpo’, ‘Ogele’, ‘Ishaoko’, ‘Scent Leaf’ and ‘Aluri’ …another one is Agbo, the leaf, with paw paw, the leaf, we add them together ….you cook it and you will be drinking it… when you deliver, you will have no problem (RAIDI/ 08).*

Many women also narrated some of the local medicines that are provided for them during labour and birth to reduce labour pains, enhance quick birth, and reduce postpartum bleeding.

*Yes, they call it ****“****ogwu adaru” …it will not allow your blood to flow when you are delivering (UA/IDI/ 07).*

Many women narrated the importance of herbal medicine and believe that, although Western medicines are effective, Western medicines may not be as potent as traditional medicines in addressing some maternal health issues. Consequently, with this belief, many women decide to use only herbal medicine when they are pregnant. Some narrate that they also combine both to get a holistic effect.

*There are some women that don’t even belief in going to facility to register for antenatal, they say no, I will not go, that this woman said I should be coming there to collect herbs, that is what will be helping me till I put to bed* (RA/IDI/ 04).

*This herbal medicine is very important because they will ask you to take these herbal medicines and not to concentrate on hospital drugs alone. They will advise you take herbal medicine, because, number one, if you don’t take it maybe when you deliver, you will have a problem (RA/IDI/ 07).*

While traditional medicine is commonly used among Igala women, many women stated that they do not use traditional medicine as it cannot be measured like Western medications and could predispose women to maternal and fetal complications, as a woman narrates.

*Local medicine doesn’t have dose and if you take over dose, it will affect you.*

*Some it will make you to be vomiting or having hot temperature or some other things, even some local medicine even forces baby to come out before labour….*

*it will cause false labour… some local medicine normally affects the liver and other things (RA/FGD/02).*

Many women indicated that, in most cases, their mothers emphasized the need for them to take herbal medicine. Women recounted that they are told that herbal medicine existed before Western medicine, and was used by their forefathers before their birth, and has helped many women navigate the route of pregnancy and childbirth. Thus, women are told that, even if they do not want to depend solely on herbal medicine, they could use both concurrently around childbirth by using Western medicine in the morning and traditional medicine in the evening or vice versa.

#### Nurturers

We explored some cultural norms within the family and communities that shape the use of MHS. The most significant factors found in this domain were family support and factors influencing the use of modern contraceptives.

#### Family support

We found that family support was both a positive and a negative nurturer in women’s use of MHS. Childbearing is an engendered role in Igalaland, where women oversee issues related to maternal health while men provide the needed finance for facility care, which was mostly how women were encouraged to use MHS.

*My husband provides all the money that I spend here during my antenatal and delivery. Because he is a very busy person, he does not come to the facility with me, but he always reminds me of the dates for my visits and also makes sure I remember to take my drugs every day (Observation 02).*

However, most women expressed their dissatisfaction with men’s engagement in maternal health, although women were happy with their husbands supporting them during labour. Many women narrated that pregnancy and childbirth was a period they could get extra money from their husbands to spend on themselves. So they were happy with the men supporting them financially and during labour. However, most women disagreed with men engaging in maternal health as they believed that would unravel the amount of money spent on facility care, which could limit their financial support and access to facility care.

*The reason I don’t like it (men’s engagement in maternal health) is that, maybe when you are coming for antenatal today, your husband follows you to that place, he will know everything that they are doing in that place, the amount they are giving card, the drugs and everything. So next time he will not give you the amount that you need again because he already knows what they are doing in that place, and before you know, problem will start. So, me I don’t like him following me (RA/FGD/02).*

Many women narrated that extended family support is important around childbirth enhancing women’s use of appropriate health facilities through financial and moral support. However, while many women welcomed their husbands’ support during labour, most women expressed dissatisfaction with some extended family members supporting them during labour and deliveries and asked for nurses to be aware of their situations.

*I don’t like it. Like my own, they came, but I was not okay with it. Because they will be seeing you going through that pain, that kind of pain, I’m not, I was not comfortable. Maybe the nurses should drive them out. The nurses should close everywhere so that they will not see me. They should just be hearing any voice (UA/IDI/06).*

However, another participant narrated that though she does not welcome extended family support in the labour room, family support outside the labour room may indicate a good relationship between a woman and the family.

*Some of them coming they are not coming eehh with good intention, some come with evil intention. I feel good, I have someone that are supporting me outside, because if as a woman if you are in the labour room and there is no one …my sister, or my sister in law …It will look like maybe am I that bad to them (RA/IDI/04).*

Some women also narrated that such family support could prolong births, and some extended family members may disclose what the woman said in the labour room to other women.

*Maybe,..God said, deliver by 2 o’clock, some that are coming there with evil mind..they may prolong the time. And some you know, we women, during labour, we are bound to say all sorts of rubbish …is not everybody that is supposed to hear what you are saying because once they are from there (labour room) they go out, they say ehhh, during labour, this is what she is saying (RA/ FGD/02).*

Some women narrated that men’s engagement in maternal health could promote their use of facility care and exhibit their support. Nevertheless, women recounted that the cultural interpretation of such support might portray a woman as a domineering and one who controls affairs in the home. Additionally, most women narrated that men engaging in maternal health could mean that such a man is stingy and engages in maternal health to pay the bill instead of trusting the wife with the payment of the bills. Based on these narratives’ most women prefer their husbands' support during labour and birth but not in antenatal care. Most women also narrated that support from extended family is crucial in women’s access to facility care. However, they emphasize the need for health workers and nurses to understand the boundaries to which such supports need to be provided. Women narrate that boundaries around childbirth are important, as they do not trust many close relations who may appear friendly, but may be harbouring plans to hurt or harm them physically or diabolically. They emphasized that such established boundaries would enhance their use of facility care.

#### Factors influencing the use of modern contraceptives

##### Ibegwu

Findings from the study showed that women had limited use of modern contraceptives due to cultural and religious structures and norms that were dominant in the communities where data was collected. In one community, many women do not openly access contraceptives because it is forbidden due to a cultural belief that such could attract the anger of Ibegwu (ancestors), which could result in the death of the husband or the male children of the family, especially when the woman in question has been married traditionally. Women are encouraged to have all the children that God has given them, as indulging in contraceptives is regarded as killing a baby. Women recount that those who indulge in family planning, especially with their husbands’ knowledge or money, would see their husbands get sick and die because of their actions. Consequently, many women in the community are afraid to use contraceptives.

*My parents, my mother inlaw all those people use to sit me down and be warning me not to do family planning…because of culture…they said that a married woman, is not good to go and do family planning, because of those Ibegwu (ancestors)…because the thing (ancestors) leads to the man’s death, being our husband. If you do family planning and your husband is aware of it the thing (Ibegwu) will lead to his death (RA/IDI/09).*

#### Male child syndrome

Many women also narrated that one of the reasons they do not access contraceptives is due to the issue of male child syndrome. In Igala land, a male child is regarded as the heir to the family's wealth as female children do not inherit family wealth since they will marry and leave the family. A woman who does not have a male child will not consent to use contraceptives as culturally, she is seen as someone who has not really produced an heir in the family.

When you don’t have a male child, you can’t go on family planning because when you go on family planning, and you need the male child….you won’t see the male child again. A male child is very important in our own tradition here because, normally if you have a female child, your husband will not be very happy with you, because the male child, when they have any talk in their family, they will call all the male children to come and sit down or all those palm trees, maybe they want to share it in your family, and you don’t have a male child, they will not give it to you, they will say you have females…. they will give it to the people that have males. Like me now, I am feeling it all the time, but I keep on praying to God (RA/IDI/08).

#### Influence of religion

Many women, both Muslims and Christians also narrated their experience of how their religion influences the use of modern contraceptives. For example, many participants explained that as Muslims, their religion does not support modern contraceptive use. A participant explained that Islam looks at using contraceptives as committing abortion, and since the Islamic faith is against abortion, they are not allowed to use contraceptives.

*Islamically, they will be preaching against it (modern contraceptives),.. because they believe doing that (using modern contraceptives) is as if you are also committing an abortion indirectly…. and Islam does not permit abortion…as if you don’t believe in your religion, you don’t believe in Allah, that the repercussion is hereafter (RA/IDI/04).*

For most Christians, women narrated that the decision on contraceptive use stays between the husband and wife on the number of children to have. However, a participant narrated that the pastors encouraged women not to use contraceptives but to continue giving birth as it is for that reason they were married.

*They will tell you to be giving birth, but don’t hide the babies in your womb; you should continue giving birth; that is why your husband married you; that is the advice they will be giving you: not to use any injection or drug (RA/IDI/02).*

However, this was not the perspective of other Christian participants, who, along with Muslim participants, narrated that the reason for the non-use of contraceptives was as a result of the side effects associated with contraceptive use, such as bleeding, secondary infertility and the need for more children, especially male children. Nevertheless, many participants related that with the present economic state in Nigeria, the use of contraceptives to achieve a small family size is crucial given the need to provide quality education and a good standard of living for the family.

## Discussion

This study sought to provide an in-depth understanding of cultural and religious structures, their meaning and how they influence Igala women’s use of MHS. These structures reflect important intergenerational norms and, in most cases, are seen as protective cultural patterns in communities. Using the PEN 3 cultural model and the focused ethnographic research gave insight into many contextual, cultural and religious structures that influence women’s use of MHS. The findings also shed light on the meanings of these structures and how such influence women’s health-seeking behaviours. Using the cultural empowerment domain of the PEN 3 cultural model, we found positive, neutral, and harmful cultural and religious structures and norms that influence women’s use of MHS. The relationship and expectation domain revealed women's perceptions and attitudes towards using culturally oriented MHS. Additionally, cultural systems and structures influencing women's use of MHS were found.

Several studies conducted in SSA countries are congruent with the current findings and showed that most of these structural factors are dominant critical issues that persistently limit women’s use of MHS and lead to high maternal and fetal mortality [5, 6]. However, using the PEN-3 cultural model also allowed us to identify a positive cultural pattern, using Indigenous language as a communication channel that could be sustained to enhance women’s use of MHS. In Igala land, community members commonly speak and understand the Igala language and Pidgin English. Our findings align with a study conducted in Tanzania, which revealed that women had a deeper understanding and use of maternal health information when using the Kiswahili native language to disseminate health information [43]. The authors also emphasize that using the Indigenous language promoted access to MHS, as seen in the present study. According to Armenta-Paulino et al. [44], language allows individuals to express their culture and social identity as language is closely linked to one’s conceptual and mental processes and how people perceive their lifeworld. Many authors have found that language barriers could result in preventable medical errors, low treatment adherence, and maternal health complications [45, 46], which could prove fatal for women. Thus, the use of Indigenous language and, if necessary, trained interpreters in maternal health education is critical, especially in rural areas with a very low literacy level. Such an approach allows women to adequately articulate their condition to enable appropriate diagnosis and management of maternal health issues.

Our study also found that spiritual and religious issues related to using prayer houses and ministries, enhancing hibernations and dependency on prayer artifacts and prophecies contributed significantly to women's non-use of MHS. Many women also emphasized that the significant reason for reliance on prayers and the use of artifacts was to seek protection from evil spirits, as pregnancy is a time when women are susceptible to attacks, which was previously discussed by Aziato et al. [8] in Ghana. However, contrary to the findings in Ghana, the authors did not state how such use of prayer artifacts influences women’s use of MHS and if financial extortion was associated with the use of such religious patterns, which was reported in this study. Given the influence of prayer houses on women’s use of MHS in Nigeria, it is critical to engage with religious leaders and proprietors of prayer houses to enhance a comprehensive approach and intervention on the way forward. Additionally, women’s spirituality reflects their dependency on the supremacy of God [8, 9], who alone is believed can orchestrate issues around childbirth. However, there should be a balance so that women do not ascribe maternal health complications as spiritual issues, diverting their attention to seeking spiritual solutions for a preventable maternal health issue, which, if not addressed in a timely manner, could have a fatal outcome.

Women’s lack of autonomy, consistent with findings in Nepal, Ghana, Tanzania and Ethiopia, was also identified in this study as a negative factor that influences access to facility care given the patriarchal system that exists in Nigeria, where the man is the decision maker in issues related to maternal health [47–50]. However, similar to the findings in Ethiopia, Zambia and Kenya, we also found that the decision to access facility care, even in emergencies, does not lie with only the husbands but with other close relations, such as the parents of the woman, the brothers and sisters [15, 17, 51, 52], delaying emergency care during emergencies that could result in birth complications. We also found that, although most women acknowledged dissatisfaction with their inability to make decisions, they do not also desire to oppose their husbands' decisions and relations to avoid being labelled as tyrants or stigmatized as one who do not conform to the traditional expectations in the community. Patriarchy is a dominant accepted cultural system in Nigeria and other parts of SSA countries that could be resistant to change. However, Kalindi et al. [52] emphasize the need for education and engagement of men in maternal health to create awareness and understanding of the need for MHS.

The use of herbal remedies during pregnancy and birth was a significant practice identified in the study, which is also consistent with findings in Kenya and Tanzania [15, 53]. The use of herbal remedies is an intergenerational practice that women have used to treat minor and significant discomfort of pregnancy and birth. However, women narrated that most herbal concoctions do not have a dosage compared to Western medicine. Most participants in the study combined both Western and native medicines, which could result in significant drug interaction that could pose a challenge for the woman and the fetus. While the Nigerian government established complementary, alternative, and traditional medicine departments in 2018 [54], there are limited enforced policies and regulations on the sale, regulation, and distribution of traditional medicine [55, 56]. Therefore, herbal medicines are made readily available to pregnant women in Nigeria without adequate proof of safety and effectiveness, predisposing most women to fake and toxic herbal medications, which could contribute to maternal and fetal morbidity and mortality [55, 57]. Appropriate enforcement of policies and regulations on the sale and distribution of herbal medicine is critical and should be accompanied by pharmaceutical evaluation to enhance understanding of the benefits of such herbal medicines on pregnancy and birth outcomes.

Family support was an important finding in the study, where women narrated husband and family support that influenced the use of MHS. Family support is also an essential component of respectful maternity care [58], which establishes a woman’s right to a companion at birth. While this scenario exists, women expressed dissatisfaction with some extended family supporting them during birth, as they believe that the period of labour and birth should be kept secret until after birth, which is also consistent with findings in Ghana, where labour and birth are kept secret to avoid witchcraft attacks that could lead to prolonged labour [48]. Women spoke about how such boundaries are essential as they do not trust most extended family members who may bewitch them and cause prolonged labour or discuss their birth experiences with other people. Consequently, most women ask health workers to be aware and maintain such boundaries during labour and birth to enhance their use of facility care around childbirth.

One of the recurrent themes in the study reflect the factors that influence the use of modern contraceptives. In both communities where data was collected, women had limited use of modern contraceptives, and one of the factors that limited the use of modern contraceptives was the palpable belief and fear of Ibegwu (ancestors), which forbids modern contraceptives and abortions and would lead to the death of a husband who agrees with the wife to use modern contraceptive [59]. This unique finding is commonly practiced in a community where data was collected for the study and is thus not applicable to other areas of Igala land. Another significant finding that influences family planning use is the issue of male child syndrome, where women do not agree to use modern contraceptives if they do not have a male child or heir in the marriage. This finding is also consistent with findings in India, where the use of contraceptives among women who had two male children was higher than those with female offspring [60, 61]. The desire for a male child in Nigeria, in most cases, prevents women from accepting modern contraceptives as male children are, in some Nigerian communities, the sole heirs to family properties [62]. These socio-cultural issues could account for the limited contraceptive prevalence of 18 percent among women aged 15- 45 years in Nigeria [59, 63, 64]. Given the low contraceptive prevalence, strategies that integrate community engagement are essential to enhance understanding of contextual and sociocultural issues, which could be harnessed [65, 66] to adequately address cultural and religious structures that impinge on the use of contraceptives.

Based on the findings of this study, the authors recommend Purnell’s Model of Cultural Competence [67]. Purnell’s Model of Cultural Competence provides a systematically organized, culturally focused assessment framework that could be applied in various settings, such as clinical practice and health systems, to promote culturally appropriate assessment and care [67, 68]. The model comprises 12 cultural components that promote assessing individuals, families and communities [67–69]. These components comprise "1. Overview, inhabited localities, and topography 2. Communication 3. Family roles and organization 4. Workforce issues 5. Biocultural ecology 6. High-risk behaviors 7. Nutrition, 8. Pregnancy and childbearing practices 9. Death rituals 10. Spirituality 11. Health care practices, and 12. Health care providers" [67, p. 99, 68, 69]. Assessment using this framework is dependent on specific cultural situations within a context [67]. Thus, many of the constructs or domains of the framework may not be used as only domains congruent with specific individual, family, community and healthcare situations are incorporated into the assessment [67, 68]. Purnell’s Model of Cultural Competence has been adapted and used in research as a theoretical framework and in different countries to propose culturally focused assessment and care [69–72]. Purnell’s model could also be adapted and used clinically and in the health system to promote culturally focused maternal health assessment and care in Nigeria and other SSA countries.

### Strengths and limitations

This study has several strengths. Our study was conducted using a focused ethnographic research methodology, allowing us to employ diverse methods to enhance a holistic and in-depth exploration of cultural and religious structures that influence women’s use of MHS in Nigeria thus yielding a thick data. Additionally, using the PEN 3 cultural model allowed us to explain the relationships between our findings and each domain of the framework to advance new knowledge on maternal health. We also purposively recruited women with diverse demographic characteristics in age, parity, location, occupation, education, and religion to enhance heterogeneous and rich findings. However, we only explored the perspectives of women using primary healthcare facilities, which may not reflect the views of women who access secondary and tertiary healthcare facilities. Thus, further research could be employed to explore the perspectives of women who access secondary and tertiary health facilities in Nigeria. Moreover, the study was conducted among an ethnic group (the Igalas) out of over 250 ethnicities in Nigeria, which necessitates future research on other ethnicities in Nigeria.

### Policy statements

The findings of the study have brought to light some of the taken-for-granted cultural issues around childbirth that need to be addressed to ensure appropriate culture-centred MHS are provided for women around childbirth. Given the dominant cultural and religious structures that influence women’s use of MHS, it is important to integrate community engagement in maternal health policies to promote an understanding of contextual and cultural issues, which could be harnessed to promote MHS provision that is responsive to communities' cultural needs [65, 66, 73]. Engaging with diverse community leaders is vital as custodians of culture and religion; leaders are role models who could encourage interventions to enhance maternal health outcomes [28, 74]. A systematic review of community engagement to improve maternal health outcomes in rural South Asia revealed women’s increased use of MHS [75]. Moreover, the realization of Millennium Development Goal (MDG) #5 in Rwanda was facilitated by intense community participation and engagement in all policies related to maternal health [76].

Additionally, introducing culturally competent nursing care in healthcare is an approach observed to be lacking in most Nigerian health facilities, where biomedical health services are merged with Indigenous cultural maternal health practices. A systematic study conducted by Jones et al. [77] explored culturally competent interventions conducted among Aboriginal and minority populations in Peru, Australia, Israel, the United States of America and the United Kingdom during pregnancy, birth and postpartum periods. The authors found that the interventions enhanced the use of MHS and were sustainable and feasible in meeting women’s cultural maternal health needs. Additionally, modules of culturally competent nursing would allow health workers and nursing students to acquire the knowledge and interpersonal skills that promote their understanding and acceptance of individuals from other cultures and others’ cultural preferences. Moreover, a nurse's cultural competence would promote inclusiveness, allowing for respect, value, and equity in care provision to ensure patients' satisfaction and safe quality care [78]. Furthermore, nurses could provide care that upholds women's dignity, essential in promoting maternal health outcomes through a continuous assessment of their values and beliefs and that of the women and community of their practices [79]. These approaches could enhance policy statements and interventions to guide the provision of appropriate culturally competent care to improve maternal health in Nigeria, enhancing the realization of the SDG goal #3:1.

## Conclusion

This study has provided an in-depth understanding and meaning of some cultural and religious structures and how they influence women’s use of MHS in Nigeria. Using the focused ethnographic research methodology and the PEN 3 cultural module has enhanced a thick description and interpretation of these cultural and religious structures. We found that using the Igala Indigenous language in health education provided engagement in health education in these contexts and enhanced women’s use of MHS. On the other hand, religious system was an existential factor that neither enhanced or limited use of MHS. However, we found that factors such as the use of prayer houses and women’s lack of autonomy limited women’s use of facility care. We also found that the use of herbal medicine is an intergenerational factor that is both a positive and negative enabler that could curb minor or major discomforts around childbirth but could also limit women’s use of MHS and lead to maternal complications. Women’s submission to cultural norms and a deep dependency on God also influenced their approach to facility care. Family support was also identified as a positive and negative nurturer that could enhance or limit women’s use of MHS. Women spoke of the need for nurses to identify boundaries regarding family support to enhance the use of MHS. Additional factors, such as Ibegwu, a dominant intergenerational factor, religion, and male child syndrome, were seen as negative nurturers that negatively influenced MHS use. Further studies are needed to understand approaches to promote appropriate culturally centred MHS that could enhance women’s use of MHS and promote the realization of SDG #3:1 in Nigeria.

## Supplementary Information

Supplementary material 1.

## Data Availability

Data are available on the reasonable request from the corresponding author.

## Abbreviations

FE: Focused ethnography
FGD: Focus group discussion
IDI: In-depth interviews
IMNCH: Integrated Maternal Newborn and Child Health Strategies
MDG: Millenium development goals
MHS: Maternal health services
MSS: Midwives service scheme
RA: Rural area
SDG: Sustainable development goals
SSA: Sub-Saharan Africa
UA: Urban area
UN: United Nations
UNICEF: United Nations Emergency Fund
WHO: World Health Organization
