# Supplementary material for: Cultural and religious structures influencing the use of maternal health services in Nigeria: a focused ethnographic research
Source: Reprod Health. 2024 Dec 18;21:188. doi: 10.1186/s12978-024-01933-8 (PMC11656583; doi:10.1186/s12978-024-01933-8)
Supplement: Supplementary file 1 — Supplementary material 1. [file 12978_2024_1933_MOESM1_ESM.docx]

**Interview Guide (One-on-One Interview)**

**Preambles**

Introduce yourself to the participant.

Describe the purpose of the interview and how information will be used.

Obtain oral consent.

Provide instructions guiding the interview.

Interview place:

Interview date:

Start time:

End time:

**Information Sheet**

| Participant Identification |  |
| --- | --- |
| Age |  |
| Number of pregnancies |  |
| Number of births |  |
| Number of children |  |
| Educational level |  |
| Occupation |  |
| Family income |  |
| Religion |  |
| Place of residence |  |

**General question:**

How has your day been today**?**

Can you tell me a little bit about yourself?

**Maternal Health Services**

**1.** Can you tell me your understanding of health services provided for women during

pregnancy and after delivery?

**2.** What are the services provided for you in this facility?

**Probes**

a. What ante-natal services are provided for you during pregnancy

b. What services are provided for you during delivery?

c. Could you tell me the services provided for you from the time of delivery to six weeks after

delivery?

**Language of Communication**

**1.** What language do health workers use to communicate with you during pregnancy and childbirth?

a. What language do health workers use to communicate with you when providing health education during antenatal and postnatal clinics?

b. In what language do health workers communicate with you when you are in labour and delivery?

2. How do health workers address women who do not understand the language of communication?

3. How does it make you feel when a particular language is used by health workers?

**Probes: a.** during health talk, **b.** during labour, **c.** during delivery, **d.** during postnatal clinic

and child immunization.

4. What do you think could be the benefit of using a particular language for communication during

health talk and around childbirth?

5. What could be the challenge of using a particular language for communication during

health talk and around childbirth?

6. How does the language used in this facility influence your use of this facility?

Probes: **a.** during pregnancy, **b.** during labour and delivery

**Influence of Religion**

1. What role does your religion play that influences your use of facility care?

2. What role does your pastor/Imam/ religious leader play to ensure that you attend antenatal care

3. What role does your pastor/Imam/ religious leader play to ensure that you deliver in a health facility?

4. What role does your pastor/Imam/ religious leader play to ensure you use a health facility after delivery?

**Influence of Prayer Houses**

**1.** Can you tell me about prayer houses and how women use them?

Probes: **a.** during pregnancy, **b.** during delivery, **c.** after delivery

2. Why do you use prayer houses?

Probes: **a.** during pregnancy, **b.** during delivery, **c.** after delivery

3. Why do you think other women visit prayer houses?

Probes: **a.** during pregnancy, **b.** during delivery, **c.** after delivery

4. What is usually done for you when you visit prayer houses?

Probes: **a.** during pregnancy, **b.** during delivery, **c.** after delivery

5. What could be the benefit of using prayer houses?

Probes: **a.** during pregnancy, **b.** during delivery, **c.** after delivery

6. What could be the challenge with using prayer houses?

Probes: **a.** during pregnancy, **b.** during delivery, **c.** after delivery

7. How does prayer house influence your use of facility care?

Probes: **a.** during pregnancy, **b.** during delivery, **c.** after delivery

**Women’s lack of autonomy**

1. How are decisions taken in your home?

2. How are decisions around pregnancy and childbirth taken in your home?

3. What is your role in decision-making around pregnancy and childbirth in your home?

4. How are decisions taken when you have an emergency?

Probes: **a.** during pregnancy, **b.** during delivery, **c.** after delivery

5. How does the community look at women who make decisions in the home?

6. How do you feel about the approach to decision-making in your home?

**Seeking Belonginess**

1. What is your opinion about cultural beliefs and practices around pregnancy and childbirth in this community?

2. What does this culture mean to you as a woman?

3. What is the benefit of respecting your culture?

4. What happens to women who do not respect the cultural norms of the community?

**Relying on the Supremacy of God**

1. How do you see God in your situation?

Probes: **a.** as a pregnant woman, **b.** as a woman going through labour and delivery, **c.**

after delivery

**Herbal Medicine**

1. Can you tell me about herbal concoctions and medicine used by you or women in this

community?

Probes: **a.** during pregnancy, **b.** during delivery, **c.** after delivery

2. What do these herbal medicines do for you?

Probes: **a.** during pregnancy, **b.** during delivery, **c.** after delivery

3. What are the benefits of using herbal medicine?

Probes: **a.** during pregnancy, **b.** during delivery, **c.** after delivery

4. What could be the problem with using herbal medicines

Probes: **a.** during pregnancy, **b.** during delivery, **c.** after delivery

5. How does using herbal medicine influence your use of facility care?

Probes: **a.** during pregnancy, **b.** during delivery, **c.** after delivery

**Family Support**

1. What role does your husband play?

Probes: **a.** during pregnancy, **b.** during delivery, **c.** after delivery

2. What role do other family members play?

Probes: **a.** during pregnancy, **b.** during delivery, **c.** after delivery

3. How would you want your husband to support you around pregnancy and childbirth?

4. How would you want other family members to support you around pregnancy and childbirth?

5. How does the support you receive influence your use of facility care?

Probes: **a.** during pregnancy, **b.** during delivery, **c.** after delivery

6. How do you feel being supported by your husband and family?

Probes: **a.** during pregnancy, **b.** during delivery, **c.** after delivery

**Factors Influencing Contraceptive Use**

1. What are the contraceptive measures you use to space childbirth?

2. What cultural factors in your community influence your use of contraceptives?

3. What is your opinion about using contraceptives?

4. How does your religion influence the use of contraceptives?

5. What are the benefits of using contraceptives as a woman?

6. What could be the problem with using contraceptives?

**Concluding question**

Is there anything more I have not said you would want to tell me?

Appreciate the participant.

End the interview.

**Guide for Focus Group Discussions**

**Preambles**

Introduce yourself to the participants.

Describe the purpose of the interview and how information will be used.

Obtain oral consent.

Provide instructions congruent with focus group discussion.

Focus group discussion context:

Focus Group discussion date:

Start time:

End time:

**Information Sheet**

| Participant Identification |  |
| --- | --- |
| Age |  |
| Number of pregnancies |  |
| Number of births |  |
| Number of children |  |
| Educational level |  |
| Occupation |  |
| Family income |  |
| Religion |  |
| Place of residence |  |

**General question:**

How has your day been today**?**

**Maternal Health Services**

**1.** Can you tell me your understanding of health services provided for women during

Pregnancy, delivery, and after delivery?

**2.** What are the services provided for you in this facility?

**Probes**

a. What ante-natal services are provided for you during pregnancy?

b. What services are provided for you during delivery?

c. Could you tell me the services provided for you from the time of delivery to six weeks after

delivery?

**Language of Communication**

**1.** What language do health workers use to communicate with you during pregnancy and childbirth?

a. What language do health workers use to communicate with you when providing health education during antenatal and postnatal clinics?

b. In what language do health workers communicate with you when you are in labour and delivery?

2. How do health workers address women who do not understand the language of communication?

3. How does it make you feel when a particular language is used by health workers?

**Probes: a.** during health talk, **b.** during labour, **c.** during delivery, **d.** during postnatal

clinic and child immunization.

4. What do you think could be the benefit of using a particular language for communication

during health talk and around childbirth?

5. What could be the challenge of using a particular language for communication during

health talk and around childbirth?

6. How does the language used in this facility influence your use of this facility?

Probes: **a.** during pregnancy, **b.** during labour and delivery

**Influence of Religion**

1. What role does your religion play that influences your use of facility care?

2. What role does your pastor/Imam/ religious leader play to ensure that you attend antenatal care

3. What role does your pastor/Imam/ religious leader play to ensure that you deliver in a health facility?

4. What role does your pastor/Imam/ religious leader play to ensure you use a health facility after delivery?

**Influence of Prayer Houses**

**1.** Can you tell me about prayer houses and how women use them?

Probes: **a.** during pregnancy, **b.** during delivery, **c.** after delivery

2. Why do you use prayer houses?

Probes: **a.** during pregnancy, **b.** during delivery, **c.** after delivery

3. Why do you think other women visit prayer houses?

Probes: **a.** during pregnancy, **b.** during delivery, **c.** after delivery

4. What is usually done for you when you visit prayer houses?

Probes: **a.** during pregnancy, **b.** during delivery, **c.** after delivery

5. What could be the benefit of using prayer houses?

Probes: **a.** during pregnancy, **b.** during delivery, **c.** after delivery

6. What could be the challenge with using prayer houses?

Probes: **a.** during pregnancy, **b.** during delivery, **c.** after delivery

7. How does prayer house influence your use of facility care?

Probes: **a.** during pregnancy, **b.** during delivery, **c.** after delivery

**Women’s lack of autonomy**

1. How are decisions taken in your home?

2. How are decisions around pregnancy and childbirth taken in your home?

3. What is your role in decision-making around pregnancy and childbirth in your home?

4. How are decisions taken when you have an emergency?

Probes: **a.** during pregnancy, **b.** during delivery, **c.** after delivery

5. How does the community look at women who make decisions in the home?

6. How do you feel about the approach to decision-making in your home?

**Seeking Belonginess**

1. What is your opinion about cultural beliefs and practices around pregnancy and childbirth in this community?

2. What does this culture mean to you as a woman?

3. What is the benefit of respecting your culture?

4. What happens to women who do not respect the cultural norms of the community?

**Relying on the Supremacy of God**

1. How do you see God in your situation?

Probes: **a.** as a pregnant woman, **b.** as a woman going through labour and delivery, **c.**

after delivery

**Herbal Medicine**

1. Can you tell me about herbal concoctions and medicine used by you or women in this

community?

Probes: **a.** during pregnancy, **b.** during delivery, **c.** after delivery

2. What do these herbal medicines do for you?

Probes: **a.** during pregnancy, **b.** during delivery, **c.** after delivery

3. What are the benefits of using herbal medicine?

Probes: **a.** during pregnancy, **b.** during delivery, **c.** after delivery

4. What could be the problem with using herbal medicines

Probes: **a.** during pregnancy, **b.** during delivery, **c.** after delivery

5. How does using herbal medicine influence your use of facility care?

Probes: **a.** during pregnancy, **b.** during delivery, **c.** after delivery

**Family Support**

1. What role does your husband play?

Probes: **a.** during pregnancy, **b.** during delivery, **c.** after delivery

2. What role do other family members play?

Probes: **a.** during pregnancy, **b.** during delivery, **c.** after delivery

3. How would you want your husband to support you around pregnancy and childbirth?

4. How would you want other family members to support you around pregnancy and childbirth?

5. How does the support you receive influence your use of facility care?

Probes: **a.** during pregnancy, **b.** during delivery, **c.** after delivery

6. How do you feel being supported by your husband and family?

Probes: **a.** during pregnancy, **b.** during delivery, **c.** after delivery

**Factors Influencing Contraceptive Use**

1. What are the contraceptive measures you use to space childbirth?

2. What cultural factors in your community influence your use of contraceptives?

3. What is your opinion about using contraceptives?

4. How does your religion influence the use of contraceptives?

5. What are the benefits of using contraceptives as a woman?

6. What could be the problem with using contraceptives?

**Concluding question**

Is there anything more I have not said you would want to tell me?

Appreciate the participants.

End the focus group discussion.
